# The Global RAdical Cystectomy Evaluation and Management (GRACEM) pathway: single‐centre prospective observational cohort study protocol

**DOI:** 10.1002/bco2.376

**Published:** 2025-01-07

**Authors:** Bruno Bernardini, Federico Piccioni, Manuele Pastore, Paolo Casale, NicolòMaria Buffi, Giovanni Lughezzani, Massimo Lazzeri, Alberto Saita, Maria Vittoria Fantacci, Stefano Mancon, Filipo Dagnino, Roberto Contieri, Pietro Brin, Stefano Mancin, Andrea Gobbo, Maria Rosaria Martucci, Giovanna Cerina, Sara Ghirmai, Ezio Lanza, Giulia Goretti, Giorgio Ferruccio Guazzoni, Rodolfoi Hurle

**Affiliations:** ^1^ Neuro‐Rehabilitation Unit, Rehabilitation Department, Neurocenter IRCCS Humanitas Research Hospital Milan Italy; ^2^ Anesthesia Unit 1, Department of Anesthesia and Intensive Care IRCCS Humanitas Research Hospital Milan Italy; ^3^ Cancer‐Center, Clinical Nutrition Unit IRCCS Humanitas Research Hospital Milan Italy; ^4^ Department of Urology IRCCS Humanitas Research Hospital Milan Italy; ^5^ Department of Biomedical Sciences Humanitas University Milan Italy; ^6^ Nursing Case Manager, Department of Urology IRCCS Humanitas Research Hospital Milan Italy; ^7^ Division of Interventional Radiology, Department of Radiology IRCCS Humanitas Research Hospital Milan Italy; ^8^ Department of Quality Management IRCCS Humanitas Research Hospital Milan Italy

**Keywords:** comprehensive geriatric assessment, frailty, geriatric syndromes, integrated clinical pathway, malnutrition, pentafecta, radical cystectomy, rehabilitation, sarcopenia

## Abstract

**Background:**

Despite guideline recommendations, few institutions have implemented clinical pathways that incorporate frailty into routine decision‐making for patients undergoing radical cystectomy (RC). This paper presents an integrated clinical pathway designed to address the needs of frail patients undergoing RC. The purpose of the study is to determine whether a multifaceted prevention programme that tailors interventions to the syndromic components of frailty can improve postoperative morbidity and recovery time for patients. New insights will be gained into how to optimize the physical and mental status and quality of life of patients before and after surgery, up to 1 year later.

**Study design:**

The Global RAdical Cystectomy Evaluation and Management (GRACEM) study is a prospective, observational, single‐centre, 2‐year cohort study. Patient enrolment began on 27 April 2023, and results are pending.

**Endpoints:**

The primary endpoints are postoperative morbidity and the in‐hospital postoperative care burden. Postoperative morbidity is measured by the number of early (up to 1 month) and late (over 1 month and up to 12 months) complications, graded by severity according to the Clavien–Dindo classification. In‐hospital postoperative care burden is measured by the number and duration of key care processes as recorded by the Care Process Monitoring Chart, a tool developed for this study. Secondary endpoints are changes in frailty and health‐related quality of life (HRQoL) from pre‐intervention to planned follow‐up up to 1 year. Frailty is assessed with the Functional Limitations and Geriatric Syndromes Frailty Questionnaire (FLIGS‐FQ), another ad hoc tool. HRQoL is assessed using the EQ‐5D‐5L questionnaire combined with the cystectomy‐specific FACT‐Bl‐cys index from the first month of follow‐up.

**Patients and methods:**

The GRACEM study includes patients with non‐metastatic, histologically confirmed, muscle‐infiltrating bladder cancer who underwent RC surgery with curative intent. This study is unique in that the GRACEM Core Team shares decision‐making throughout the pathway, from before the intervention to the end of the patient's follow‐up. The pathway involves the patient, family members and community services.

## 1 BACKGROUND

Radical cystectomy (RC) with pelvic lymphadenectomy and urinary diversion is the cornerstone of curative treatment for non‐muscle‐invasive bladder cancer at high risk of recurrence and progression and for non‐metastatic muscle‐invasive bladder cancer.^1^ RC is a technically challenging procedure that carries a high burden of morbidity, mortality and poor quality of life for patients.^2^ The complication rate at 30 and 90 days after RC surgery can be as high as 80%, and the mortality rate at 30 and 90 days is 2.1% and 4.7%, respectively.^3^

Over the past two decades, surgery has been transformed by the introduction of Enhanced Recovery After Surgery (ERAS) protocols into clinical practice. ERAS protocols define evidence‐based multimodal interventions for key time periods in the surgical pathway that have become the standard of care for all patients undergoing major surgery. Incorporating many of the recommendations from colorectal surgery, the ERAS Society published the first guidelines for bladder cancer patients in 2013.^4^

Adherence to ERAS standards in RC surgery has been shown to have a beneficial impact on patients compared to conventional care. Several meta‐analyses have demonstrated a compelling effect size in the reduction of time‐dependent postoperative indicators (e.g. return of bowel function to regular diet, length of hospital stay), compared with substantial stability in cancer‐related indicators such as morbidity, mortality and readmission rates.^5^, ^6^, ^7^, ^8^ However, the large heterogeneity in time‐related indicators suggests that patients differ in their ability to comply with ERAS standards due to poorly considered and managed features such as geriatric syndromes typical of older patients.

Advanced age, functional dependence, cognitive impairment, comorbidities including malnutrition and sarcopenia and frailty have been found to be variably associated with adverse outcomes after RC surgery, to the point of being candidates as independent predictors.^9^, ^10^, ^11^ Despite evidence^12^ and recommendations in many guidelines to include comprehensive geriatric assessment (CGA) and frailty as routine components to optimize the clinical management of patients undergoing surgery,^13^, ^14^, ^15^, ^16^, ^17^ few institutions have successfully implemented this practice, and almost none for RC.^18^, ^19^, ^20^

In this article, we present the Global RAdical Cystectomy Evaluation and Management (GRACEM), a multidisciplinary care pathway,^21^, ^22^ developed for patients undergoing RC surgery at our institution. The GRACEM pathway is based on frailty and geriatric syndromes as key elements for individualizing diagnostic, therapeutic and rehabilitation decisions. The unique aspect of the GRACEM pathway is that the Core Team (case manager, urologist, anaesthesiologist, geriatrician, clinical dietitian, nurse and rehabilitation professionals) shares and coordinates all decisions at all stages of the pathway, from preoperative to hospital discharge and follow‐up, with active patient and family involvement.^23^

In essence, the GRACEM pathway states that the assessment of frailty at baseline, both in its syndromic components and as a whole, guides the optimization of the patient's condition before surgery and helps to decide on the surgical strategy commensurate with his or her vulnerability. The same baseline assessment is also used to proactively plan postoperative care and anticipate discharge scenarios. During the 1‐year follow‐up, a worsening of the patients' frailty indicates the need for increased intensity of care, including rehabilitation.

Once the perioperative safety of the GRACEM pathway has been demonstrated, this study will have three objectives:

- To determine whether the multifaceted prevention programme, tailored to individual frailty, has a protective effect on the occurrence of early complications and on the quality and recovery time of patients in the postoperative period.
- To identify the best strategies to optimize patients' physical and mental status and health‐related quality of life before surgery and during the 1‐year follow‐up period.
- To identify a core set of indicators and measures that best predict outcomes and allow stratifying patients according to frailty risk.

## 2 STUDY DESIGN

The GRACEM pathway study is a prospective, observational, single‐centre cohort study with 1‐year patient follow‐up. Patient enrolment began on 27 April 2023 and will end on 30 April 2025. Approximately 40 patients will be enrolled annually. Data collection will reach completion in April 2026. For ethical reasons, the pathway is open to all patients in need of RC. However, only patients who meet the inclusion criteria will be enrolled in the study.

Ethical approval was obtained from the independent ethics committee of the IRCCS Humanitas Research Hospital (N° 3317; 18/11/2022). Written informed consent is obtained from all patients at the time of inclusion in the GRACEM pathway.

### 2.1 Setting

The GRACEM pathway is the result of collaboration between the urology and Neurorehabilitation Units and reflects our current practice. The Urology Unit is a 40‐bed unit dedicated to the treatment of all surgical conditions of the genitourinary system. For RC, ERAS protocols have been implemented since 2014 and open surgery with uretero‐ileo‐cutaneostomy (Bricker) or orthotopic ileal neobladder is the standard.

The Neurorehabilitation Unit is a 30‐bed unit dedicated to the multimodal rehabilitation of acute‐subacute patients with neurological and neurosurgical disabilities, including cancer patients. In addition to its core activities, the team provides neuro‐geriatric consultation and rehabilitation treatments to all departments of the hospital to prevent functional decline in inpatients and to appropriately transition more complex cases from the hospital to community services.

IRCCS Humanitas Research Hospital is a Joint Commission International (JCI)‐accredited tertiary teaching hospital. Since January 2016, an integrated electronic health record (IEHR) has been used to track healthcare provider activities and promote compliance with JCI standards.^24^ Patient‐centred JCI standards address access and continuity of care, patient assessment, care delivery, safety goals, anaesthesia and surgical care, medication management and patient and family rights and education. They are a good framework for incorporating specific pathways, such as the GRACEM pathway.

### 2.2 Frailty assessment

Disability, comorbidity and psychosocial issues underlie the construct of most multidimensional frailty assessment tools. However, existing tools use different taxonomies and metrics, so there is no consensus on a gold standard for clinical use.^25^, ^26^

In designing the GRACEM pathway, we felt that keeping the measures of the main components of CGA separate might be more useful for communication across disciplines and for guiding individualized treatment decisions. Therefore, we developed an ad hoc frailty screening tool based solely on the patient‐reported burden of functional limitations and geriatric syndromes while considering comorbidities, including malnutrition and sarcopenia, and psychosocial concerns considered as separate domains of frailty.

The Functional Limitation and Geriatric Syndromes Frailty Questionnaire (FLIGS‐FQ) is a patient‐reported measure that we are using in the GRACEM study both as a screening tool and as a health outcome measure during patient follow‐up (see Supporting Information).

## 3 ENDPOINTS

The primary endpoints are postoperative morbidity and in‐hospital postoperative care burden. Postoperative morbidity is measured by the number of early (up to 1 month) and late (more than 1 month and up to 12 months) complications, categorized by severity according to the Clavien–Dindo classification (CDC).^27^ Intra‐hospital complications are tracked in the IEHR. Out‐of‐hospital complications are collected in a standardized manner by the GRACEM case manager at 15 days and at 1, 6 and 12 months after patient discharge and graded for severity by an assessor outside the team. In‐hospital postoperative care burden is measured by the number and duration of key care processes recorded using the Care Process Monitoring Chart (CPMC) (see Section 5.9). Due to the nature of the study and the use of the IEHR, blinding cannot be guaranteed.

Secondary endpoints are the changes in frailty and HRQoL from pre‐intervention to follow‐up at 1, 6 and 12 months. Frailty is assessed using the FLIGS‐FQ, while HRQoL is assessed using the generic EQ‐5D‐5L questionnaire^28^ combined with the cystectomy‐specific FACT‐BI‐cys index^29^ from the 1‐month follow‐up.

## 4 ELIGIBILITY CRITERIA

The GRACEM study enrols patients with histologically proven, muscle‐infiltrating, non‐metastatic bladder cancer who undergo RC surgery with curative intent. Inclusion in the study of patients with concomitant tumours is evaluated on a case‐by‐case basis by the Core Team.

The following patients are excluded from the GRACEM study, but not from the pathway:

- Patients undergoing salvage RC for refractory haematuria or severe symptoms,
- Patients for whom the time between indication for surgery and RC is too short to allow a complete baseline assessment,
- Patients for whom the intent of the RC surgery has changed from curative to palliative after a complete baseline evaluation.

## 5 METHODS

### 5.1 The GRACEM pathway

**FIGURE 1:**
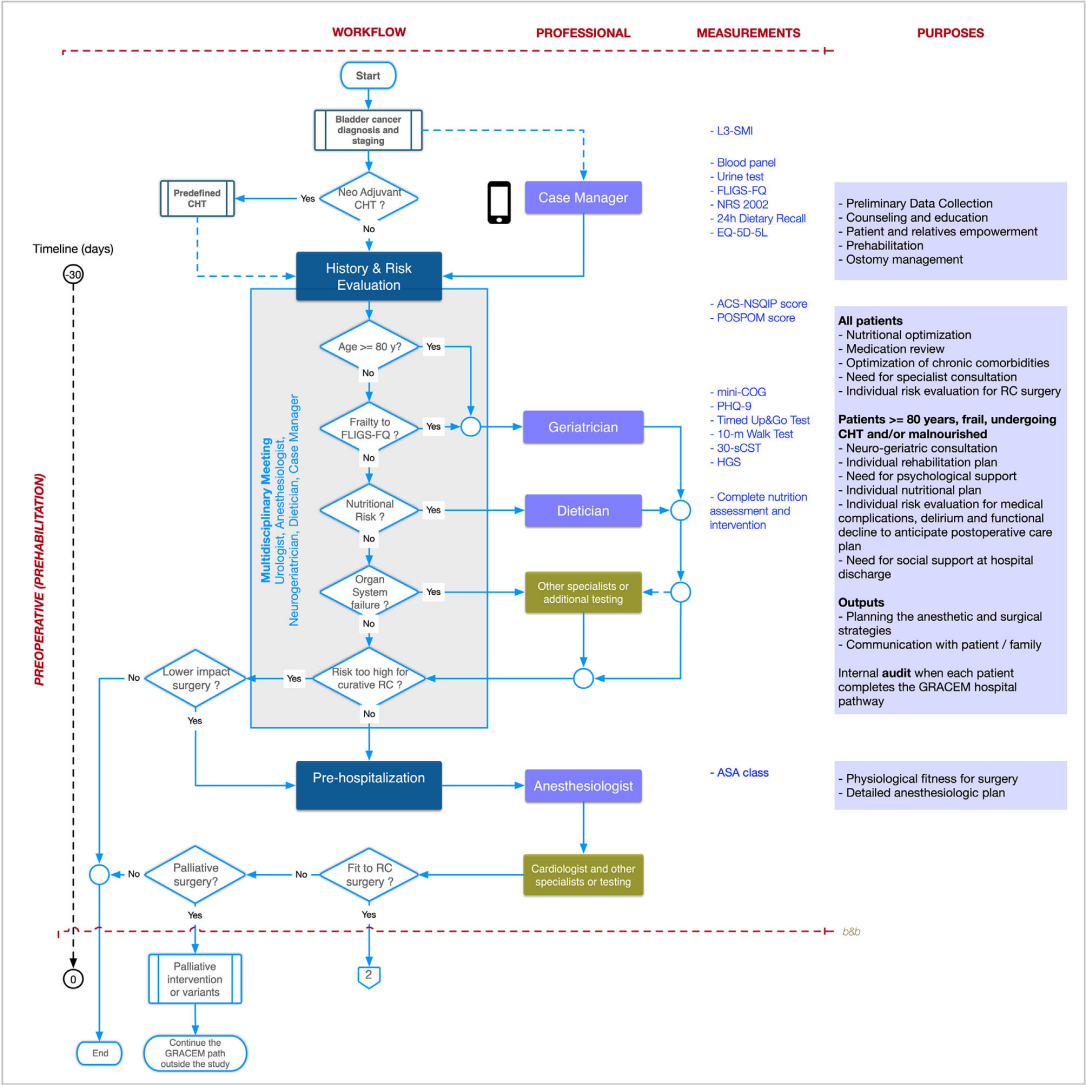
Map of preoperative decision‐making episodes. 30‐sCST, 30‐second chair‐stand test; ACS‐NSQIP American College of Surgeons National Surgical Quality Improvement Program; ASA class, American Society of Anesthesiologist classification; EQ‐5D‐5L, EuroQol Group EQ‐5D; FLIGS‐FQ, Functional Limitation and Geriatric Syndromes Frailty Questionnaire; HGS, hand grip strength; L3‐SMI, L3 Skeletal Muscle Index; mini‐COG, mini‐Cognitive quick screening for dementia; NRS‐2002, Nutritional Risk Screening‐2002; PHQ‐9, Patient Health Questionnaire‐9; POSPOM, Preoperative Score to Predict Postoperative Mortality.

**FIGURE 2:**
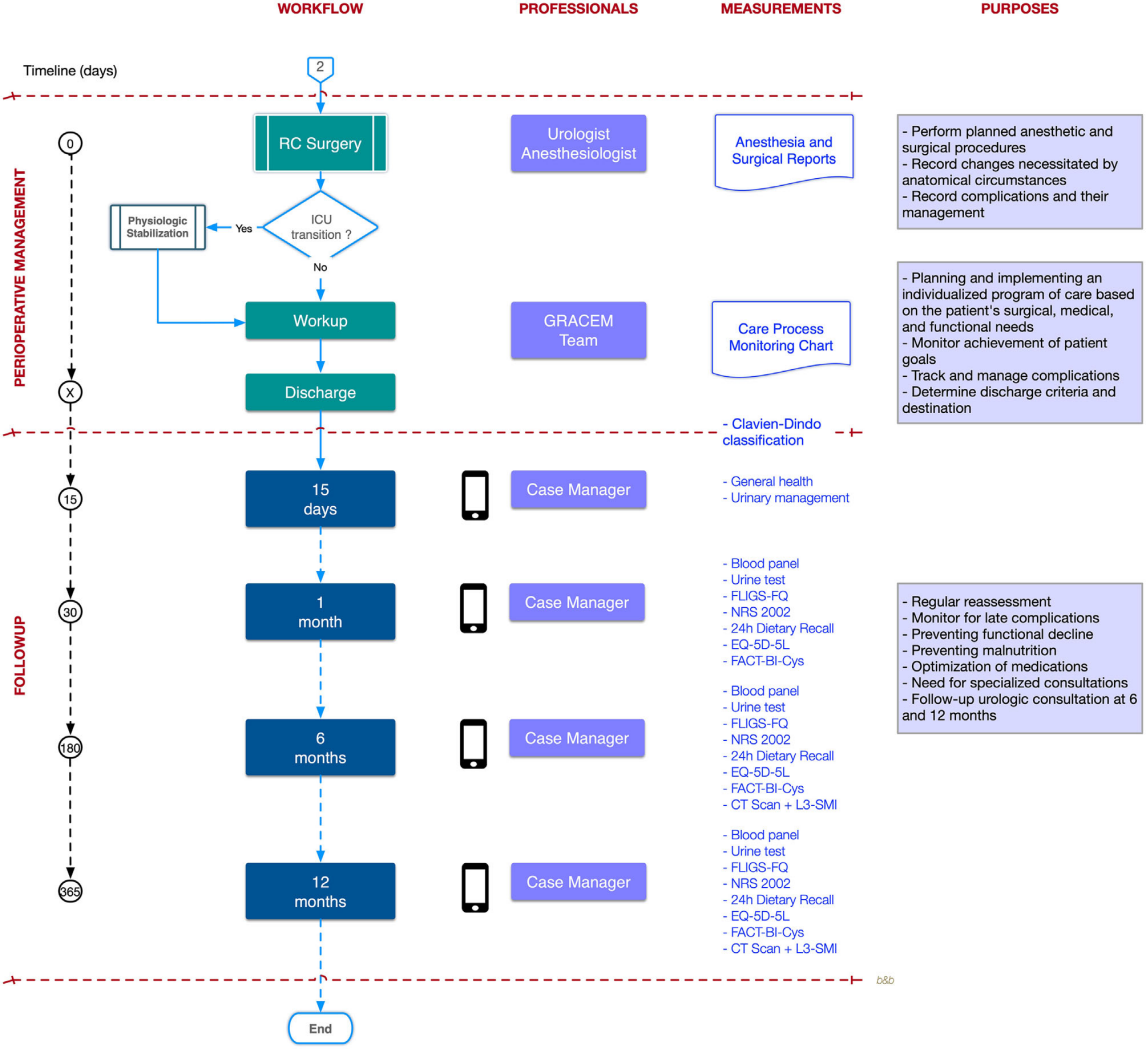
Map of perioperative and follow‐up decision‐making episodes. EQ‐5D‐5L, EuroQol Group EQ‐5D; FACT‐Bl‐Cys, Functional Assessment of Cancer Therapy‐Bladder‐Cystectomy; FLIGS‐FQ, Functional Limitations and Geriatric Syndromes Frailty Questionnaire; L3‐SMI, L3 Skeletal Muscle Index; NRS‐2002, Nutritional Risk Screening‐2002.

The GRACEM pathway is outlined for key diagnostic‐therapeutic decision‐making events during the Core Team workflow in the preoperative (Figure 1) and postoperative (Figure 2) steps. Best practices recommended by clinical guidelines are indicated in the text and are assumed to be followed appropriately.

During or immediately after bladder cancer staging, patients requiring RC are referred by their urologist to the case manager for enrolment in the GRACEM pathway. Staging CT of the abdomen and pelvis is used to assess at the L3 level the cross‐sectional area of the lumbar muscle and determine sarcopenia by calculating the Skeletal Muscle Index (L3‐SMI). Patients receiving neoadjuvant chemotherapy are enrolled at the end of the cycle and all undergo neuro‐geriatric consultation.

At least 1 month before the scheduled RC, the case manager contacts patients by telephone and administers the FLIGS‐FQ to assess frailty. He also asks patients to complete the Nutritional Risk Screening (NRS‐2002),^30^ the 24‐h Recall^31^ and the EQ‐5D‐5L^28^ to assess nutritional status, food intake, and HRQoL, respectively. The case manager also collects basic anthropometric data (habitual weight, current weight, height, BMI) and requests blood tests (blood glucose, glycated haemoglobin, triglycerides, total cholesterol, HDL, LDL, Ca, Cl, Na, Mg, K, creatinine, urea, AST, ALT, GGT, albumin, prealbumin, total protein, iron, ferritin, transferrin, CBC, PT, PTT, 25‐OH vitamin D, vitamin B12, CRP and ESR) and urinalysis.

Once all documentation is available, patients are included in the weekly multidisciplinary meeting.

### 5.2 Pre‐habilitation

Pre‐habilitation in the GRACEM pathway involves a two‐tiered approach: the first, led by the case manager for all patients, and the second, individually planned after neuro‐geriatric and/or nutritional consultations for patients with frailty and/or undernutrition–sarcopenia or obesity.

In preparation for the intervention, the case manager provides patients with basic education on how to modify high‐risk lifestyle behaviours (e.g. stop smoking, reduce alcohol consumption, reduce sedentary time). Patients are strongly encouraged to follow an aerobic and resistance training programme at home that is appropriate for their functional status and level of physical activity. The case manager's recommendations are given verbally and through iconographic materials during a preliminary face‐to‐face visit. During this visit, the principles necessary postoperative ostomy self‐management are also outlined.

### 5.3 Multidisciplinary meeting

The objectives of the first GRACEM Team meeting are to jointly plan the surgical and anaesthesia strategies, to anticipate the perioperative care and the outcome at discharge and to establish appropriate communication with the patient and family. These goals cannot be achieved without the best possible understanding, albeit empirical, of the risk–benefit ratio for each patient with respect to early and late postoperative outcomes.^32^

As a starting point and guide for discussing patient risk during the initial GRACEM Team meeting, we use the American College of Surgeons' National Quality Improvement Program in Surgery calculator (ACS‐NSQIP; http://riskcalculator.facs.org). The risk of mortality is further estimated using the Preoperative Score to Predict Postoperative Mortality (POSPOM).^33^

The review of information during the first meeting of the GRACEM Team is the main opportunity to optimize the patient's health problems before surgery based on the pathological or borderline elements revealed by the history, laboratory tests, FLIGS‐FQ and nutritional assessment, including sarcopenia. All comorbidities are reviewed, nutritional deficits are corrected, additional tests are ordered, or other specialists are consulted if deemed necessary. Patient‐reported medication use is reviewed for appropriateness of therapy and accuracy of dosing.^34^

In addition to patients who have completed neoadjuvant chemotherapy, candidates for RC who are 80 years of age or older, or who are frail according to the FLIGS‐FQ, and/or who are malnourished or obese, are considered at “high risk” and referred for neuro‐geriatric and/or nutritional consultation.

Alternatively, patients younger than 80 years of age who are not frail according to the FLIGS‐FQ are not malnourished or obese, and do not have severe organ failure (e.g. cardiac, respiratory, renal, hepatic, dementia) are considered at ‘usual risk’ and are referred to the prehospital step without further evaluation. All patients with severe organ failure are referred for specialist consultation.

### 5.4 Neuro‐geriatric consultation

The neuro‐geriatrician conducts the examination guided by the qualitative items reported on the FLIGS‐FQ, which are further explored with a focused history and physical and functional examination. Special attention is given to items attributable to underlying neurological disorders (e.g. dizziness and falls, dysphagia, memory loss and sensory disturbances) that have prognostic value for serious perioperative outcomes such as pulmonary aspiration, delirium and functional decline.

If the patient lives alone and has no close family members, the strength of parental support and the social context are assessed, as psychosocial frailty has a negative impact on the patient's resilience and sustainability of care. In the case of social frailty, our hospital's Continuing Care Service is proactively alerted.

During the examination, the mini‐COG^35^ (https://mini-cog.com/) and the PHQ‐9^36^ are administered to screen for dementia and depression, respectively; the Timed‐Up‐and‐Go Test (TUG)^37^ and 10‐m walk speed are measured for motor performance; and the 30‐s chair stand test (30‐sCST) and handgrip strength (HGS)^38^ are measured as clinical correlates of sarcopenia.^39^

### 5.5 Dietician consultation

The goal of dietary counselling is to optimize nutrition prior to surgery by assessing parameters such as anthropometric measurements, nutritional needs, dietary habits, biochemical tests, clinical conditions and lifestyle. Specific dietary recommendations improve patients' food choices and correct nutritional imbalances. When diet alone proves insufficient, specific nutritional supplements may be recommended. In some situations, a detailed, individualized diet plan may be necessary. If oral feeding is not feasible, enteral or parenteral nutrition may be considered, following relevant guidelines.^40^

At the end of their consultations, both the neuro‐geriatrician and the dietitian may adjust therapy, order additional tests, request consultation with other specialists or prescribe individualized rehabilitation. For example, the neuro‐geriatrician may refer the patient to the physiotherapist for a tailored rehabilitation programme for modifiable impairments, to the speech therapist for evaluation and treatment of dysphagia or speech disorders, to the neuropsychologist for formal evaluation if previously unknown dementia is suspected, to the psychologist for emotional support in cases of depressive complaints or to the psychiatrist for treatment of major disorders.

### 5.6 Patient–family communication and engagement

The results of the initial multidisciplinary meeting provide a joint understanding of the patient's early and late risks and the most appropriate treatment strategies to reduce them.

The urologist, or the entire team in more challenging cases, meets with the patient and family/caregivers to discuss the benefits and risks of curative RC compared with other less invasive options, the risk of complications, the prospect for special supportive therapies and the expected outcome of the entire pathway to foster informed choice and consent to the planned treatments.^23^ This balanced communication takes place in the context of the patient's quantity and quality of life and takes into account the patient's expectations and preferences.

Patients for whom curative RC is not feasible and for whom surgery may be palliative will still be offered surgical treatment. As mentioned above, they will be excluded from the GRACEM study but not from the pathway. Patients considered to be at very high risk (e.g. failure to thrive, severe organ failure, including dementia) and/or with unexpected cancer progression may be excluded from surgery. If they are terminally ill, they will be referred to home palliative care or hospice.

### 5.7 Prehospitalization

The prehospital step focuses, as usual, on the examination of organ systems to assess the patient's physiologic fitness for surgery, particularly the cardiovascular, respiratory and renal systems.

Prior to the visit, the anaesthesiologist already has a good understanding of the patient's frailty, comorbidities, nutritional status, functional status and medications, which are useful in refining risk‐based anaesthesia planning after the history and focused physical examination.^41^ At the end of the visit, anaesthesia risk is coded using the ASA classification.^42^

All information is provided to the cardiologist for guideline‐directed cardiac risk assessment and management.^43^ The anaesthesiologist and cardiologist may order additional examinations or tests or involve other specialists to better define previously unrecognized problems or complex therapies, such as those for coagulopathies.

### 5.8 Anaesthesia and surgical intervention

Many of the ERAS procedures, including avoidance of prolonged preoperative fasting and preanaesthesia sedatives, antibiotic, antiemetic and venous thromboembolism prophylaxis, use of opioid‐sparing analgesic techniques based on thoracic epidural analgesia, maintenance of normothermia, optimization of haemodynamics with cardiac output monitoring and goal‐directed strategy, monitoring of depth of anaesthesia with bispectral index and neuromuscular blockade and protective mechanical ventilation, are implemented through comprehensive evidence‐based standards of care.^41^

The surgery is performed as planned. The urologist's operative report includes total operative time, procedural difficulties or deviations, any complications and how they were managed.

### 5.9 Postintervention management

Current best practice guidelines are followed for the management of postoperative medical and surgical issues. The institutional Acute Pain Service Team provides a multimodal approach to postoperative pain control and minimization of opioid use and medication‐related adverse effects.^44^

All patients are started on a normal diet on the day of surgery unless contraindicated. Patients previously identified as being at risk for malnutrition are given high‐protein oral nutritional supplements. Enteral or parenteral nutrition is started early in malnourished patients according to current guidelines.^45^ At discharge, malnourished, sarcopenic or frail patients are prescribed high‐protein oral nutritional supplements.

Early mobilization is provided to all patients by the physiotherapist. Patients with dysphagia and/or delirium or cognitive disorders are evaluated and treated by the speech therapist and/or neuropsychologist. For postoperative delirium,^46^ internal procedures are in place to guide pharmacologic, behavioural and environmental management, including the continuous presence of a family member at the patient's bedside to reduce the use of physical restraints and prevent fall.

To objectify and measure the complex network of care processes provided by the GRACEM Team, we developed the CPMC by incorporating surgical risk indicators, including some ERAS items, into a set of medical and functional risk indicators already used in the Neurorehabilitation Unit.^47^

Data collection in the CPMC during daily care allows measurement of the time it takes each patient to achieve the goals of complete medical stability (i.e. absence of medical risk indicators), autonomy in eating a normal diet and independence in basic mobility (i.e. getting out of bed and walking more than 3 m).

### 5.10 Discharge

The decision to discharge a patient is not based solely on the stability of surgical outcomes. The CPMC easily highlights the co‐occurrence of residual medical and functional problems and thus serves as a checklist to support the appropriateness of the timing of transfer and the safety of the post‐hospital care environment.

Patients with surgical outcomes deemed optimal, no indicators of medical risk, independence in normal diet and basic mobility are discharged home with family support only. Patients with residual minor medical and/or functional problems are discharged home with activation of home health and/or physiotherapy services. Patients with residual major medical and/or functional problems requiring multimodal management are transferred to an intensive or extensive rehabilitation or long‐term care facility. At discharge, the IEHR is reviewed to obtain a critical overview of the patient's course and to assess complications.

### 5.11 Follow‐up

Follow‐up appointments are scheduled at 15 days and at 1, 6 and 12 months after the patient is discharged from the hospital. At each telephone contact after discharge, the case manager detects complications according to a standardized form.

At 15 days, the case manager conducts a telephone interview with the patient and caregiver to obtain information about health status and problems related to urostomy management.

The case manager's contact with the patient or caregiver at 1, 6 and 12 months aims to obtain the same assessment as at baseline plus the FACT‐BI‐Cys^29^ index to assess specific HRQoL after RC.

The results of each reassessment are discussed by the GRACEM Team according to a plan that follows the initial team meeting. After grading of complications, nutritional status and medications are optimized, and further examinations or specialist visits are requested if necessary. Recommendations for appropriate physical activity are reinforced. Patients with worsening of frailty (i.e. new geriatric syndromes or new functional limitations) and/or nutritional status are referred for neuro‐geriatric and/or dietary consultation, either in person or via telemedicine.

At the 6‐month and 12‐month follow‐up CT scans of the pelvis, abdomen and chest, the sarcopenia measurements will be repeated, and a follow‐up urologic consultation will be performed. Throughout the follow‐up period, the GRACEM Team remains available for urgent consultations.

## 6 METHODS OF DATA COLLECTION

Except for the Frailty and HRQoL questionnaires and external examinations, all other GRACEM pathway data are routinely recorded in the patient's IEHR, including multidisciplinary meetings, specialist consultation reports, laboratory tests and imaging procedures.

Using Research Electronic Data Capture (REDCap) tools,^48^ a database of all relevant data from the GRACEM pathway was created and will be maintained in accordance with the purposes of the study.

## 7 ANALYSIS PLAN

We planned to have an independent statistician perform an interim safety analysis of the frailty screening process 1 year after the start of data collection. In fact, the first preoperative GRACEM Core Team meeting arbitrarily stratifies patients by frailty into two risk subgroups, ‘high’ and ‘usual’, who are referred for advanced or standard prehospital evaluation, respectively. Comparing these two groups for in‐hospital primary endpoints may reveal a disproportionately high false‐negative rate in frail patients, impacting perioperative safety and necessitating a revised screening process.

To evaluate the effects of the GRACEM programme in terms in‐hospital morbidity and care burden, a historical group of patients who underwent RC between January 2016 and December 2019 and met the same inclusion criteria will be used as a control group. These data will be collected retrospectively by the IEHR. The end of the inclusion period in 2019 was chosen because of the COVID‐19 pandemic, which may have compromised the normal course of healthcare in Italy.

Mixed models will be used to examine longitudinal changes from baseline in FLIGS‐FQ scores for frailty and in the EQ‐5D‐5L^28^ and FACT‐Bl‐cys^29^ for HRQoL.

Data analysis will be performed using STATA/SE, version 18.0 (StataCorp). We will consider two‐sided *P* ≤ 0.05 to be statistically significant.

## 8 DISCUSSION

The design of clinical pathways for major cancer surgery must take into account individual risk factors to ensure appropriate access to care, modulate surgical strategies according to patient prognosis and preferences, provide proactive, non‐iatrogenic perioperative care and ensure adequate and safe discharge. Personalization of pathways is critical for frail, often elderly patients who have competing risk factors with cancer‐specific ones that can be minimized by integrating rehabilitation strategies with surgical care.

Current models of care for cancer surgery emphasize pre‐habilitation and adherence to ERAS standards as the only critical components for successful surgery in a one‐size‐fits‐all manner and without differentiation among patients. Thus, important gaps remain in understanding how to integrate geriatric risk factors with cancer‐specific risk factors for preoperative stratification of patients, how to establish and monitor tailored perioperative care plans and how to determine optimal outcome measures for time, quality of recovery and patient acceptance.

The GRACEM pathway, with its streamlined and easily replicable integration between the specific routines of the surgical and rehabilitation teams, provides a comprehensive mapping of patient co‐management across the clinical continuum. The pathway measures meet all standards recommended by major guidelines for the perioperative care of elderly patients undergoing elective major surgery.^49^, ^50^, ^51^

Well‐designed studies to improve the quality of cystectomy care already exist.^52^ However, to our knowledge, the GRACEM pathway is the first to address and operationalize at the patient's bedside the multiple diagnostic and therapeutic challenges posed by frail patients undergoing radical cystectomy.

Assessment of frailty for its components of disability and geriatric syndromes initiates the patient's GRACEM pathway. Subsequently, markers of chronic comorbidities, including malnutrition, sarcopenia, dementia and social frailty, are incorporated into interdisciplinary reasoning to modulate diagnostic and therapeutic decisions and optimize the patient's health status prior to RC surgery. After RC surgery, continuous monitoring of key markers of the medical, surgical and functional care processes is used to guide optimal recovery and to create a patient‐specific goal attainment performance profile to support the discharge decision.

At discharge, this allows us to identify the composite outcome of ‘successful recovery’ for the patient, a kind of clinical‐functional pentafecta that meets the following criteria: discharge home, no medical or surgical problems requiring activation of home services, autonomy in communication, eating normal meals and basic mobility. This pentafecta may complement the similar composite oncology outcomes of the RC.^53^

In addition to traditional measures of healthcare quality, the GRACEM pathway includes three patient‐reported outcome measures: the FLIGS‐FQ, the EQ‐5D‐5L and the FACT‐Bl‐Cys. These tools add important information about the impact of RC surgery on the health and experience of the patient and can help to improve the decision‐making approach of the GRACEM pathway. Once safety and clinical utility have been demonstrated, the GRACEM pathway may become a standard that can be adapted for other types of major surgery in our institution.

This study has several limitations. It is a single‐centre study, highly context specific and subject to bias because the investigators and outcomes assessors are not blinded. Therefore, the GRACEM study can serve as a reference for other institutions similar to ours. However, its results cannot be generalized.

## AUTHOR CONTRIBUTIONS

*Study design*: Hurle Rodolfo, Bernardini Bruno, Piccioni Federico and Lazzeri Massimo. *Review of relevant literature to fit the clinical pathway: Rehabilitation and frailty*, Cerina Giovanna, Ghirmai Sara and Bernardini Bruno; *Anaesthesia*, Piccioni Federico and Martucci Maria Rosaria; *Urology*, Bernardini Bruno, Lughezzani Giovanni, Mancin Stefano, Dagnino Filippo, Contieri Roberto, BF and Gobbo Andrea; *Nutrition*, Pastore Manuela and Mancin Stefano. *GRACEM pathway design*: Bernardini Bruno and Goretti Giulia. *Drafting*: Bernardini Bruno, Hurle Rodolfo and Piccioni Federico. *Manuscript revision for important contents*: Lazzeri Massimo, Pastore Manuela, Mancin Stefano and Guazzoni Ferruccio. *Coordination of clinical activities*: Hurle Rodolfo, Fantacci Maria Vittoria, Piccioni Federico and Pastore Manuela. *Participation in clinical activities of the GRACEM pathway*: All authors. All authors read and approved the final version of the manuscript.

## CONFLICT OF INTEREST STATEMENT

All authors declare that they have no competing interests.

## Supporting information


**Data S1.** Supporting Information
