# Supplementary material for: The Global RAdical Cystectomy Evaluation and Management (GRACEM) pathway: single‐centre prospective observational cohort study protocol
Source: BJUI Compass. 2025 Jan 7;6(1):e376. doi: 10.1002/bco2.376 (PMC11771505; doi:10.1002/bco2.376)
Supplement: Supplementary file 1 — Data S1. Supporting Information [file BCO2-6-e376-s001.docx]

Supplements to the research protocol

“The Global Radical Cystectomy Evaluation and Management (GRACEM) Pathway: Single-Center Prospective Observational Cohort Study Protocol

Bernardini Bruno, Piccioni Federico, Pastore Manuela, et al.

IRCCS Humanitas Research Hospital,

Via Alessandro Manzoni 56, 20089 Rozzano, Milan, Italy.

**L3 Skeletal Muscle Index (L3-SMI) ^1,2^**

**Functional Limitations and Geriatric Syndromes (FLIGS) Frailty Questionnaire (ver. 2.1a)**

| Items |  | |  | | |  | |
| --- | --- | --- | --- | --- | --- | --- | --- |
| 1. FUNCTIONAL LIMITATIONS | | No | | Yes | Notes | | |
| 1. Is supported by a caregiver or relative for more than 6 hours a day | |  | |  |  | |  |
| 1. Must be followed or helped to bathe or shower | |  | |  |  | |  |
| 1. Needs help getting dressed | |  | |  |  | |  |
| 1. Is he/she supervised or helped in moving around the home | |  | |  |  | |  |
| 1. Needs help to manage medications | |  | |  |  | |  |
| 1. Needs frequent supervision in usual activities | |  | |  |  | |  |
| 1. Must always be accompanied when it is necessary to leave the house | |  | |  |  | |  |
| 1. Must use a cane or other aid to walk outside the home | |  | |  |  | |  |
| Subscore | | | | 0-8 |  | |  |
|  | | | |  |  | |  |
| 1. GERIATRIC SYNDROMES | | No | | Yes |  | |  |
| 1. Has frequent dizziness or balance problems. | |  | |  |  | |  |
| 1. Has serious vision problems | |  | |  |  | |  |
| 1. Has severe hearing problems or wears hearing aids | |  | |  |  | |  |
| 1. Takes 5 or more medications per day (excluding supplements and vitamins) | |  | |  |  | |  |
| 1. Has major memory problems | |  | |  |  | |  |
| 1. Has fallen in the last 6 months | |  | |  |  | |  |
| 1. Has difficulty swallowing or often coughs when drinking | |  | |  |  | |  |
| 1. Lost a lot of weight in the past 6 months | |  | |  |  | |  |
| 1. Often feels down or depressed | |  | |  |  | |  |
| 1. Has incontinence problems and use pads to avoid getting wet | |  | |  |  | |  |
| 1. Suffers from insomnia | |  | |  |  | |  |
| 1. Takes sedatives or sleeping pills | |  | |  |  | |  |
| 1. Often complains of pain | |  | |  |  | |  |
| 1. Often feels weak and fatigued | |  | |  |  | |  |
| 1. Has behavior problems | |  | |  |  | |  |
| Subscore | | | | 0-15 |  | |  |
|  | | | |  |  | |  |
| FLIGS-FQ Total score | | | | 0-23 |  | |  |

| 1. SOCIAL FRAILTY * | No | Yes | Notes | |
| --- | --- | --- | --- | --- |
| 1. He is being followed by social services |  |  |  |  |
| 1. Lives alone |  |  |  |  |
| 1. He has no relatives or friends who are able to assist and care for him |  |  |  |  |

* Social frailty items are not included in the FLIGS-FQ score.

The FLIGS-FQ collects the patient's history over the past 30 days to screen for functional limitations in activities of daily living and geriatric syndromes. The FLIGS-FQ consists of 23 dichotomous (false/true) questions that can be easily administered by trained nonspecialist staff during a face-to-face or telephone interview with the patient or caregiver, or self-administered. The average compilation time of FLIGS-FQ is 8 minutes (range 5-11).

The FLIGS-FQ produces a continuous score ranging from 0 (no frailty) to 23 ("failure to thrive"), with a cut-off score of 2 separating non-frailty individuals from progressively more frail individuals. Preliminary psychometric validation results confirmed the dimensionality and consistency of the FLIGS-FQ construct and the good reliability of the score.

In addition, we found that the total score of the FLIGS-FQ was associated with the incidence of adverse clinical events occurring during hospitalization in a sample of elderly patients admitted from the emergency department to the internal medicine ward (Bernardini B., Pedale R., Lemorini G., De Iaco L., Arosio P., et al. Manuscript in preparation).

**Preoperative Score to Predict Postoperative Mortality (POSPOM)** ^3^

| ITEMS | Score | Age | Score |
| --- | --- | --- | --- |
| Chronic heat failure or cardiomyopathy | 4 | 51-55 | 7 |
| Hemiplegia | 4 | 56-60 | 8 |
| Chronic alcohol abuse | 4 | 61-65 | 9 |
| Chronic respiratory failure | 3 | 66-70 | 10 |
| Dementia | 2 | 71-75 | 11 |
| Transplanted organs | 2 | 76-80 | 12 |
| Chronic renal failure | 2 | 81-85 | 13 |
| Ischemic heart disease | 1 | 86-90 | 14 |
| Cardiac arrythmia or heart blocks | 1 | 91-95 | 15 |
| Peripheral vascular disease | 1 | 96 + | 16 |
| Cerebrovascular disease | 1 |  |  |
| Chronic obstructive lung disease | 1 |  |  |
| Diabetes | 1 |  |  |
| Preoperative chronic hrmodialysis | 1 |  |  |
|  |  | Total score | _______ |

**Neurogeriatric Consultation form**

History

Chronic multimorbidities

Functional

Family /social support

Physical Examination

Medications

Conclusion

| **mini-COG scoring** ^4^ | | |  | **Patient Health Questionnaire (PHQ-9)** ^5^ | | | | |
| --- | --- | --- | --- | --- | --- | --- | --- | --- |
|  |  |  |  |  |  |  |  |  |
| Three Word Recall |  | (0 – 3 points) |  | Score |  |  |  |  |
|  |  |  |  |  |  |  |  |  |
| Clock Draw |  | (0 or 2 points) |  | 5 - 9 | mild |  |  |  |
|  |  |  |  | 10 - 14 | moderate | | | |
| Total score |  | (0 – 5 points) |  | 15 - 19 | moderately severe | | | |
|  |  |  |  | >20 | Severe |  |  |  |

| **Timed Up&Go (TUG) Test** ^6^ | | |  | **10-meter Walk Test** (10-mWT) ^7^ | | | | |
| --- | --- | --- | --- | --- | --- | --- | --- | --- |
|  |  |  |  |  |  |  |  |  |
|  |  | seconds |  | m/s |  |  |  |  |
|  |  |  |  |  |  |  |  |  |
| It should be considered that factors such as gender, height, weight, and pathological conditions can affect individual performance. The test results should be interpreted by an experienced healthcare professional. | | | | | | | | |
|  |  |  |  |  |  |  |  |  |

**30-seconds Chair Stand Test (30s-CST)** available at https://www.cdc.gov/steadi/pdf/STEADI-Assessment-30Sec-508.pdf

|  | Age (y) | | | | | | |
| --- | --- | --- | --- | --- | --- | --- | --- |
|  | 60-64 | 65-69 | 70-74 | 75-79 | 80-84 | 85-89 | 90-94 |
|  |  |  |  |  |  |  |  |
| Men | < 14 | < 12 | < 12 | < 11 | < 10 | < 8 | < 7 |
| Women | < 12 | < 11 | < 10 | < 10 | < 9 | < 8 | < 4 |

**Hand Grip Strenght (HGS) ^8^**

|  | Dominant  Hand (Kg) | Non Dominant Hand (Kg) |  |
| --- | --- | --- | --- |
| Test 1 |  |  |  |
| Test 2 |  |  |  |
| Test 3 |  |  |  |
| Best score |  |  |  |

|  |  | Age (years) | | | | | | | | |
| --- | --- | --- | --- | --- | --- | --- | --- | --- | --- | --- |
|  | Male | 50 y | 55 y | 60 y | 65 y | 70 y | 75 y | 80 y | 85 y | 90 y |
| Centiles (kg) | 90 th | 60 | 59 | 56 | 53 | 49 | 45 | 42 | 38 | 33 |
|  | 75 th | 54 | 53 | 51 | 48 | 44 | 41 | 37 | 33 | 29 |
|  | 50 th | 48 | 47 | 45 | 43 | 39 | 35 | 32 | 29 | 25 |
|  | 25 th | 41 | 40 | 39 | 37 | 34 | 31 | 27 | 24 | 20 |
|  | 10 th | 35 | 34 | 33 | 31 | 29 | 26 | 23 | 19 | 16 |
|  |  |  |  |  |  |  |  |  |  |  |
|  | Female |  |  |  |  |  |  |  |  |  |
| Centiles (Kg) | 90 th | 37 | 35 | 34 | 33 | 31 | 28 | 26 |  |  |
|  | 75 th | 33 | 32 | 31 | 29 | 27 | 25 | 23 |  |  |
|  | 50 th | 29 | 28 | 27 | 25 | 24 | 21 | 19 |  |  |
|  | 25 th | 25 | 23 | 22 | 21 | 20 | 18 | 16 |  |  |
|  | 10 th | 21 | 19 | 18 | 17 | 16 | 14 | 13 |  |  |

**Care Process Monitoring Chart (CPMC)**


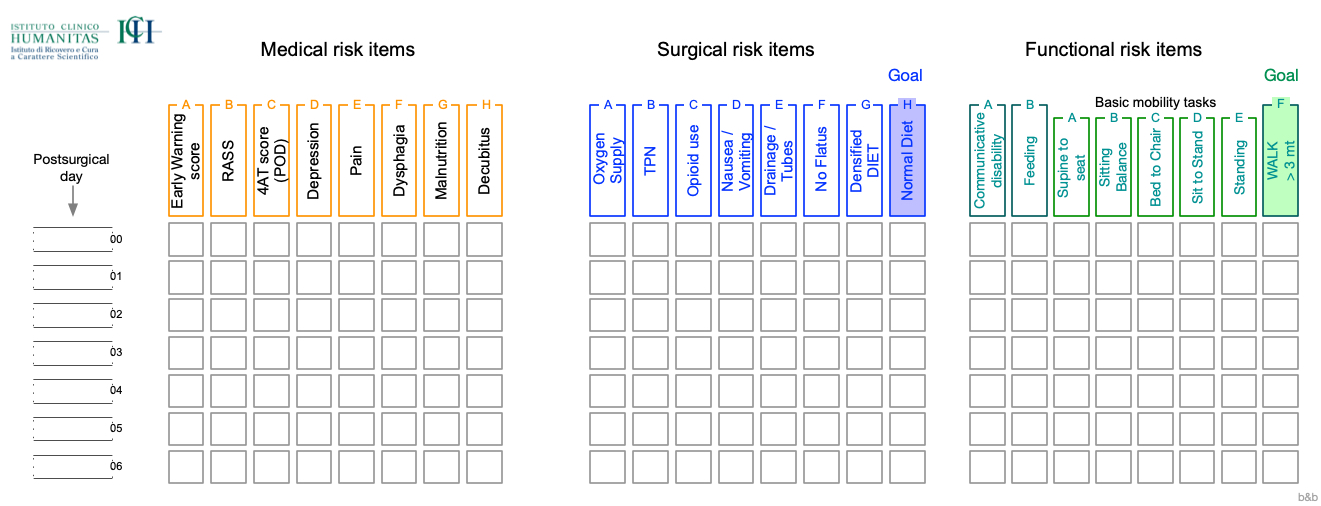


Abbreviations: EWS, Early Warning Score;^9^ RASS, Richmond Agitation-Sedation Scale;^10^ 4AT, 4 “A” Test screening for delirium.^11^

Although they are complications, postoperative delirium (POD) and ileus (Flatus) are reported as key processes in CPMC because of their importance. Delirium will be reported as a major complication regardless of its duration. All other complications will be classified according to the Clavien-Dindo classification.

**Definition of medical an functional items (from the IPER-2.0 system)** ^12^

1. Depressed Mood-Depression

- Target: presence of depressed mood or depression

- Algorithm: If the patient shows signs of depressed mood or depression AND/OR is taking antidepressant drugs, THEN YES (1), ELSE NO (0).

Note: The presence of Reduced Alertness or Delirium makes the detection of Depression unreliable. If the patient is able to communicate administer depression screening questionnaires. If communicative disability is present be alert for non-verbal signs of depression.

2. Pain

- Target: presence of pain (somatic and/or neuropatic).
- Algorithm: IF the patient reports significant pain (Numeric Rating Scale >3/10), OR the physical exam indirectly shows signs of pain (think for example, grimacing in pain in people who are unable to communicate their feelings), THEN YES (1), ELSE NO (0).

3. Dysphagia
- Target: presence of dysphagia
- Algorithm: IF the patient shows signs of dysphagia on the 3 oz water swallow test or is fed through a nasogastric tube or PEG, THEN YES (1), IF NO (0).

4. Malnutrition

- Target: presence of malnutrition

- Algorithm: IF the patients show physical (e.g. loss of muscle mass, Body Mass Index <18.5) AND laboratory signs of malnutrition (e.g. hypoalbuminemia), THEN YES (1), ELSE NO (0).

5. Pressure Sore

- Target: presence of pressure sore
- Algorithm: If the patient has one or more pressure ulcers at stage ≥S2 (AHCPR classification) in any part of the body THEN YES (1), ELSE NO (0).

## Markers of Funtional Dependence

This subset of indicators explores three areas of functional dependence: in communication, in eating, and in performing six key tasks of basic mobility (getting out of bed, transferring to a chair, and walking at least 10 feet).

1. Communicative Disability

- Target: presence of moderate to severe communicative disability
- Algorithm: If the patient shows a moderate to complete communicative disability (Communicative Disability Scale score 2 to 4), THEN YES (1), ELSE NO (0).

| COMMUNICATIVE DISABILITY SCALE (CDS) | **Burden of Communication** |
| --- | --- |
| 1. None: Reports your history reliably and is able to discuss all personal issues. Well related to the interlocutor, participates without problems in the maneuvers of physical examination and care. | **100% Patients** |
| 1. Mild: He should be "guided" to retrieve the history and stimulated to talk about personal issues. Adequate to the interlocutor but it is necessary to adopt a directive attitude in order to obtain full participation in the maneuvers of physical examination and care. |  |
| 1. Moderate: The information is only exchanged by asking simple questions for a strictly personal and actual communicative context. Adequate to the interlocutor but cannot to provide a satisfactory participation in the maneuvers of physical examination and care. |  |
| 1. Severe: Communication is very compromized and information can only be obtained with questions that involve yes/no answers and by providing contextual and gestural facilitations. Perplexed in the relationship with the interlocutor and does not participate in the maneuvers of physical examination and care. |  |
| 1. Complete: Almost no communicative exchange, even with gestural facilitations. Not adequate for the interlocutor and absolutely not participative in the maneuvers of physical examination and care. | **100% Caregiver** |

**2. Dependence in Eating**- Target: dependence in eating.

- Algorithm: If the patient is unable to eat by himself OR someone must actively assist him/her during the meal (Barthel Index^13^ subscore Alimentation ≤2) THEN YES (1), ELSE NO (0).

**3. Dependence on Supine to Seated Transfer**- Target: dependence on supine-to-seated transfer.
- Algorithm: If the patient is unable OR needs physical assistance in transferring from supine to sitting position, THEN YES (1), ELSE NO (0).

**4. Dependence on Sitting Balance**- Target: dependence on sitting balance.
- Algorithm: If the patient is unable OR needs physical assistance in maintaining the sitting position for at least 30 second, THEN YES (1), ELSE NO (0).

**5. Dependence in Bed-to-Chair Transfer**- Target: dependence on the bed-to-chair transfer.
- Algorithm: If the patient is totally unable OR needs physical assistance to transfer from bed to chair (armchair), THEN YES (1), ELSE NO (0).

Barthel Index,^13^ subscore bed to chair transfer: <8.

**6. Dependence in Sit-to-Stand**- Target: dependence to stand up from a chair.
- Algorithm: If the patient is unable OR needs physical assistance to get up from a chair and achieve the standing position THEN YES (1), ELSE NO (0).

**7. Dependence in Standing**- Target: dependence on standing position
- Algorithm: IF the patient is unable OR needs physical assistance to stand without support (open eyes, any position of the feet) for at least 30 seconds, THEN YES (1), ELSE NO (0).

**8. Dependence on Walk (>3 meters)**- Target: dependence on walking at least 3 meters.
- Algorithm: If the patient is unable OR needs physical assistance to walk at least three meters, THEN YES (1), ELSE NO (0).

The use of walking aids (e.g., cane, tripod, walker) is permitted.

**Follow-up interview form**

| Patient Id. |  |  |  |  |
| --- | --- | --- | --- | --- |
|  |  |  |  |  |

| Date | Followup | Interview with | Where he lives now and with whom |
| --- | --- | --- | --- |
|  |  15 days |  Patient |  |
|  |  1 month |  Family member/caregiver |  |
|  |  6 months |  Responsible Physician |  |
|  |  12 months |  Other |  |

| Weight (kg) |  |  |  |  |
| --- | --- | --- | --- | --- |
|  |  |  |  |  |

| **Lifestyle** | **Yes** | **No** | **Notes** |
| --- | --- | --- | --- |
| Drinks at least one and a half liters of water daily |  |  |  |
| Takes prescribed alkalizers |  |  |  |
| Drinks blueberry juice |  |  |  |
| Takes nutritional supplements, if prescribed |  |  |  |
| Leaves home for the daily walk |  |  |  |
| Practice exercise 3 times a week for at least 30 min. |  |  |  |
| Has returned to work (if applicable) |  |  |  |

| **Trouble with** | **No** | **Yes** |  |
| --- | --- | --- | --- |
| Appetite for foods |  |  |  |
| Bowel |  |  |  |
| Wound |  |  |  |
| Urostomy |  |  |  |
| Urine |  |  |  |

| **Complications** | **No** | **Yes** | **When** | **For what reason** |
| --- | --- | --- | --- | --- |
| 1.a Needed a visit to the family GP |  |  |  |  |
| 1.b |  |  |  |  |
| 1.c |  |  |  |  |
| 2.a Had to take antibiotics |  |  |  |  |
| 2.b |  |  |  |  |
| 2.c |  |  |  |  |
| 3.a Had to modify drug therapy |  |  |  |  |
| 3.b |  |  |  |  |
| 3.c |  |  |  |  |
| 4.a He had to go to the hospital * |  |  |  |  |
| 4.b |  |  |  |  |
| 4.c |  |  |  |  |
| 5.a He was hospitalized * |  |  |  |  |
| 5.b |  |  |  |  |
| 5.c |  |  |  |  |
| Death * |  |  |  |  |

*In case of hospital episodes acquire documentation

References

1. Portal D, Hofstetter L, Eshed I, et al. L3 skeletal muscle index (L3SMI) is a surrogate marker of sarcopenia and frailty in non-small cell lung cancer patients. *Cancer Manag Res*. 2019;11:2579-2588. doi:10.2147/CMAR.S195869

2. Lanza E, Masetti C, Messana G, et al. Sarcopenia as a predictor of survival in patients undergoing bland transarterial embolization for unresectable hepatocellular carcinoma. *PLoS One*. 2020;15(6):1-12. doi:10.1371/journal.pone.0232371

3. Froehner M, Koch R, Hübler M, et al. Validation of the Preoperative Score to Predict Postoperative Mortality in Patients Undergoing Radical Cystectomy. *Eur Urol Focus*. 2019;5(2):197-200. doi:10.1016/j.euf.2017.05.003

4. Covinsky KE, Pierluissi E, Story THEPS. CLINICIAN ’ S CORNER Hospitalization-Associated Disability “ She Was Probably Able to Ambulate , but I ’ m Not Sure ”. 2014.

5. Levis B, Benedetti A, Thombs BD. Accuracy of Patient Health Questionnaire-9 (PHQ-9) for screening to detect major depression: Individual participant data meta-analysis. *BMJ*. 2019;365. doi:10.1136/bmj.l1476

6. Podsiadlo D RS. The Timed “Up&Go”: a test for basic functional mobility for frail elderly persons. *J Am Geriatr Soc*. 1991;39(2):142-148.

7. Tyson, S; Connel L. The psychometric properties and clinical utility of measures of walking and mobility in neurological conditions: a systematic review. *Clin Rehabil*. 2009;23(11):1018-1033.

8. Dodds RM, Syddall HE, Cooper R, et al. Grip strength across the life course: Normative data from twelve British studies. *PLoS One*. 2014;9(12):1-15. doi:10.1371/journal.pone.0113637

9. Royal College of Physicians. National Early Warning Score (NEWS) 2 Standardising the assessment of acute-illness severity in the NHS Updated report of a working party Executive summary and recommendations The Royal College of Physicians. 2017;(2017):3.

10. Ely EW, Truman B, Thomason JWW, et al. Monitoring sedation status over time in ICU patients. Reliability and Validity of the Richmond Agitation-Sedation Scale (RASS). 2003;289:2983-2991.

11. Bellelli G, Morandi A, Davis DHJ, et al. Validation of the 4AT, a new instrument for rapid delirium screening: A study in 234 hospitalised older people. *Age Ageing*. 2014;43(4):496-502. doi:10.1093/ageing/afu021

12. Bernardini B, Baratto L, Pizzi C, et al. A multicenter prospective study validated a nomogram to predict individual risk of dependence in ambulation after rehabilitation. *J Clin Epidemiol*. 2023;154:97-107. doi:10.1016/j.jclinepi.2022.10.021

13. Shah, S; Vanclay, F; Cooper B. Improving the sensitivity of the Barthel Index for stroke rehabilitation. *J Clin Epidemiol*. 1989;42(8):703-709.
